# The role of gastrostomy in the staged operation of esophageal atresia

**DOI:** 10.4103/0971-9261.42565

**Published:** 2008

**Authors:** Seyed Mohammad Vahid Hosseini, Sam Zeraatian Nejad Davani, Babak Sabet, Hamid Reza Forutan, Maryam Sharifian

**Affiliations:** Department of Pediatric Surgery, Shiraz University of Medical Sciences, Shiraz, Iran

**Keywords:** Congenital heart disease, esophageal atresia, gastrostomy, tracheoesophageal fistula

## Abstract

**Introduction:**

The aim of this study is to recommend criteria for selection of patients who benefited from the use of gastrostomy rather than emergency fistula closure during the staged operation of esophageal atresia (EA).

**Materials and Methods:**

Between August 2004 and July 2006, 75 cases of EA, were consecutively operated. Nineteen out of 75 (25%) underwent routine gastrostomy because they required a type of staged operation: Group I: Five cases with pure atresia had gastrostomy and esophagostomy; Group II: Six with severe pneumonia and congenital heart disease (Waterson class C) had gastrostomy and conservative management; Group III: Eight with long gap EA (2-4 vertebras); four out of 8 cases underwent primary anastomosis with tension and the other four had delayed primary anastomosis plus primary gastrostomy.

**Results:**

GI: Only three cases survived after esophageal substitution; GII: Three out of six cases with severe pneumonia (fistula size: f > 2.5 mm) underwent emergency fistula closure with only one survival, but all (f < 2.5 mm) recovered without complication, GIII: Four patients with long gap and primary anastomosis with tension developed anastomotic leakage; they required gastrostomy following the leakage, except for those with delayed primary anastomosis, and all of them recovered without early complications.

**Conclusion:**

All the cases with long gap, although two esophageal ends can be reached with tension, should undergo delayed primary closure with primary gastrostomy. Those were brought with Waterson class C and the fistula size greater than 2.5 mm should undergo emergency fistula closure; however, if fistula size was less than 2.5 mm, it is better to be delayed by primary gastrostomy for stabilization. In this study, we had a better outcome with gastric tube for substitution than colon interposition in infants.

## INTRODUCTION

Esophageal atresia (EA) correction is regarded as the symbol of neonatal surgical expertise, while the evolution of EA treatment from simple ideas to staged and robotic operation has elapsed over 60 years.[1–2] Surgical options depend on the condition of patients and suitability of native esophagus; although advancing neonatal care and operative techniques are available, even premature babies with severe pneumonia who are not stable for thoracotomy are now undergoing thoracotomy for fistula closure.[3]

The mortality of thoracotomy in poor conditioned babies is very high therefore, the use of guidelines regarding those who may benefit from gastrostomy for delayed or staged operation, instead of performing thoracotomy for emergency fistula closure in the first step, is very helpful.

## MATERIALS AND METHODS

Between August 2004 and July 2006, we have retrospectively studied 75 cases of consequently operated EA (24 females, 51 males) from their files. Nineteen out of 75 cases (25 %) underwent routine gastrostomy in the left upper quadrant with mushroom No. 20 F (5 cases with pure atresia, six cases with severe pneumonia and congenital heart disease and 8 cases with long gap 2-4 vertebra). They were staged or delayed by gastrostomy for following definitive repair [Table 1].

**Table 1:** Distribution of esophageal atresia patients who underwent primary gastrostomy

EA with G tube (19 out of 75 cases)	Primary gastrostomy	Emergency fistula closure	Primary anastomosis	Delayed anastomosis	Staged operation
Three groups					
Pure atresia (5 cases)	5	No	No	No	3 cases with gastric pull-up. 2 cases with colon interposition
Severe pneumonia and CHD Waterson class C (6 cases)	6	3 cases with f > 2.5 mm	3 cases with f < 2.5 mm (3-7 ds)	One survival in emergency fistula closure	No
Long gap (8 cases, 2-4 vertebras)	4	No	4 cases with high tension	4	No
Results	15	2 patients died after closure	4 cases with anastomotic leakage, 4 cases with post-leakage gastrostomy	1 case with severe reflux	2 patients with colon interposition died

### Group I:

Five cases of pure atresia first underwent gastrostomy and cervical esophagostomy and then gastrostomy for esophageal atresia esophageal substitution (3 cases with gastric pull-up and 2 cases with colon interposition) was performed 9-12 months later.

### Group II:

Six out of 75 cases (8%) - who had severe pneumonia, weight (1700-2000 g) and (Waterson class C) - underwent primary gastrostomy and conservative therapy that includes the use of mechanical ventilation (*n* = 6) and antibiotic and cardiac management in an acceptable ICU setting. The size of the fistula was measured by pass telescopic lens (∼2.5 mm) preoperatively by bronchoscopy. Three patients who had wide fistula (fistula size >2.5 mm) presented within 48 hours with severe distention of abdomen and paradoxical abdominal movements. However, for those with a narrow fistula, the arrival was at more than 48 h post-delivery; there was respiratory distress and tachypnea with sticky bilious discharge from the endotracheal tube (ETT). Three out of 6 cases (Spitz class II), who had congenital heart disease in addition to severe pneumonia (1 case with TOF, 2 cases with large VSD) and a wide fistula (f > 2.5 mm) required emergency fistula closure on the second or third day. One of emergency closures was performed by injecting the fibrin glue through bronchoscope using double lumen catheter No. 7 as the Y connector. The closure of fistula was checked by the injection of methylene blue into the ETT, and in the others, the fistula was closed through thoracotomy. The remaining 3 cases with f < 2.5 mm were subjected to primary anastomosis in 3-7 days through right thoracotomy, and they were followed for 6-12 months.

### Group III:

Eight out of 75 cases (10%) had long gap (2-4 vertebra); four cases underwent right extra pleural thoracotomy and excessive dissection of two ends, which were approximated by traction suture (monofilament non absorbable) plus primary gastrostomy for feeding, and they had delayed primary anastomosis 2-3 months later. The other four cases had primary anastomosis, but with a high tension without *primary gastrostomy*. All cases were followed-up for 6-12 months.

## RESULTS

Nineteen out of 75 cases with EA and a mean gestational age of 36.8 weeks (34-38 weeks) and weight in the range of 1700-3200 g were subjected to gastrostomy and a type of staged operation.

### Group I:

All the five cases in this group survived till the time of substitution. Only 3 gastric pull-up cases survived from definitive operation, and the site of gastrostomy made no trouble during pull-up procedure. Two colon interposition cases died of postoperative leakage and sepsis.

### Group II:

Two out of six (33%) cases (severe pneumonia and congenital heart disease) with f > 2.5 mm, who developed massive air leakage through gastrostomy that prevented effective ventilation, died early after the emergency fistula closure and the remaining one underwent thoracotomy and anastomosis 3 months later with no early or late complications. Three out of six patients with severe pneumonia and f < 2.5 mm had no bilious discharge after gastrostomy and their condition improved considerably. After having undergone primary anastomosis, all of them recovered with no major early postoperative complications (mean hospitalization of 15 ± 3 days), and all weaned off the ventilator in a matter of 2-3 days postoperatively. Two out of four (50%) survived cases (f < 2.5 mm) developed anastomotic stricture, and they underwent repeated dilatation in the follow-up for 6-12 months.

### Group III:

Four cases that had primary anastomosis in the first operation developed anastomotic leakage. They all were conservatively managed by gastrostomy and jejunostomy following the leakage (mean hospitalization of 45 ± 2 days). Gastrostomy was used for the prevention of reflux to the site of anastomotic leakage. Two out of four cases developed late anastomotic strictures and there was one case of fistula recurrence in 6-12 months follow-up. The remaining four cases of long gap had delayed primary closure with primary gastrostomy. In the second operation, the small and atrophic distal segment developed so well, and this allowed the two ends be anastomosed safely with no major complications (mean hospitalization of 18 ± 3 days). Only 1 out of 4 delayed primary anastomosis cases developed severe reflux and repeated pneumonia who underwent partial fundoplication in the follow-up for 6-12 months [Table 1].

## DISCUSSION

In this study, 13 out of 19 (68%) patients with TEF had excellent outcomes after primary gastrostomy (*n* = 15), and they have reached the staged operation, except for the two cases with severe pneumonia and CHD. The roles of gastrostomy in patient with pure atresia and control of anastomotic leak are obvious, and we have just overviewed our series of patients. However, in those with long-gap atresia, particularly those with an intermediate gap (2-5 vertebras) and those who referred with unstable conditions, are still the subjects of debate on whether they should undergo delayed or staged operation.

Mee *et al*.[4] have designed classifications for the priority of management such as Waterston[5] and Spitz;[6] however, the most important factors to be considered are the significance of congenital heart disease, severity of pneumonia and gestational age. We have seen in our study that the highest mortality belongs to the patients with CHD and pneumonia who have associated larger fistulas (f > 2.5 mm) because they cannot tolerate the invasive operation in spite of pneumonia and unstable cardiac condition.

Based on our experience, all the patients with narrower fistula have higher chance of reflux into tracheobronchial tree and chemical pneumonitis, particularly with the use of ventilator or late diagnosis. It can be explained by the capillary movement through the narrow fistula with help of negative pressure in the thoracic cavity and gradual positive pressure built in stomach, which siphon the gastric content to tracheobronchial tree; however, the patient with larger fistulas develop respiratory compromise earlier because larger fistula can causes severe distension of the stomach and ineffective ventilation before developing reflux pnemonitis. The use of primary gastrostomy, even in small fistula during mechanical ventilation, helps in the evacuation of stomach and further reflux. Therefore, patients with Waterson class C (3 of 6 cases with f < 2.5 mm) have delayed (range: 3-7 ds) definitive operation while patients are stabilized, instead of staged operation.

Emergency fistula closure has been recommended for babies who develop respiratory failure despite the maximum intervention and compliance and the easy passage of respiratory gases through the distal fistula into the stomach requires close monitoring for the possible risk of gastric perforation.[7] In our cases, the size of the fistula was a determinant factor. In those who referred with respiratory compromise, whenever the fistula diameter is less than the diameter of trachea (premature, term/2.5, 3 mm), the volume of ventilated gas preferably goes through the major airway, and gastrostomy works as a decompressing valve and prevents bile reflux into the tracheobronchial tree and improves diaphragmatic movement. In a larger fistula, all the air bypasses the tracheobronchial tree via gastrostomy and the effective ventilation is lost. In the absence of gastrostomy, severe abdominal distension or gastric perforation occurred because even the manipulation the ETT cannot prevent the massive air leakage through a large fistula. Therefore, large fistulas (f >2.5 mm) should be closed as soon as possible.

Touloukian *et al*.[8] have recommended that a rigid bronchoscope should be used in all the neonates with EA to precisely detect the location, size and number of any associated fistulae and the degree of possible tracheomalacia. However, we performed bronchoscopy on our patients in the preoperative period whenever there was a need for a respiratory toilet and temporary closure of fistula using Fogarty balloon or glue injection in suspicious cases.

If a long gap is found during thoracotomy, several options have been proposed: the two ends of the esophagus may be put under tension by the steel bars, stringed beads or magnets and in a matter of 5-6 days, two ends can be anastomosed.[9–10] However, the fiber damages from overstretching the esophageal muscle, perforation or mediastinitis from suture detachment and dysmotility of the repaired esophagus are common complications.[9]

Bagolan *et al*.[10] have also reported that if there is a delay in definitive operation, let the distal segment develop by the reflux content. In our opinion, a delay of 2-3 months allows the esophagus to grow and the reflux content make the narrow distal segment well developed, particulary with high volume gastrostomy feeding and a dissection of lower segment that can disrupt all the antireflux mechanism. Therefore, inflammation by the irritation of gastric content can be the cause of distal overgrowth.

The comparison of early and late complications indicates reflux in the delayed primary anastomosis group; however, in early leakage, the chances of stricture (50%) and recurrence (25%) are much higher in the group with primary high-tension anastomosis, although two ends can be reached by traction.

## CONCLUSION

Because of high rate of postoperative complications in the group with long gap and high-tension anastomosis, the use of gastrostomy is highly recommended with delayed primary anastomosis. Patients who presented with pneumonia and CHD (Waterston class C) need preoperative bronchoscopy for the evaluation of the fistula. If fistula size is greater than 2.5 mm, then patient should undergo emergency closure, and if the fistula size is less than 2.5 mm, they need primary gastrostomy for stabilization. We also had better outcomes with gastric tube than colon during esophageal substitution.

## References

[1] Myers NA (1974). Esophageal atresia: The epitome of modern surgery. Ann R Coll Surg Engl.

[2] Ashcraft KW, Holder TM (1969). The story of esophageal atresia and tracheo-esophageal fistula. Surgery.

[3] Eradi B, Narasimhan KL, Rao KL, Grover A, Samujh R, Chowdhary SK (2003). Waterston's classification revisited: It relevance in developing countries. J Indian Assoc Pediatr Surg.

[4] Mee RB, Beasley SW, Auldis AW (1992). Influence of congenital heart disease on management of esophageal atresia. Pediatr Surg Int.

[5] Waterston DJ, Carter RE, Aberdeen E (1962). Oesophageal atresia: Tracheo-oesophageal fistula: A study of survival in 218 infants. Lancet.

[6] Spitz L, Kiely EM, Morecroft JA, Drake DP (1994). Oesophageal atresia: At-risk groups for the 1990s. J Pediatr Surg.

[7] Maoate K, Myers NA, Beasley SW (1999). Gastric perforation in infants with esophageal atresia and distal tracheo-esophageal fistula. Pediatr Surg Int.

[8] Touloukian RJ, Seashore JH (2004). Thirty-five-year institutional experience with end-to-side repair for esophageal atresia. Arch Surg.

[9] Kimura K, Nishijima E, Tsugawa C, Collin DL, Lazer EL, Stylianous S (2001). Multistaged extrathoracic esophageal elongation procedure for long gap esophageal atresia: Experience with 12 patients. J Pediatr Surg.

[10] Foker JE, Linden BC, Boyle EM, Marquardt C (1997). Development of a true primary repair for the full spectrum of esophageal atresia. Ann Surg.

